# Auxin regulation involved in gynoecium morphogenesis of papaya flowers

**DOI:** 10.1038/s41438-019-0205-8

**Published:** 2019-11-01

**Authors:** Ping Zhou, Mahpara Fatima, Xinyi Ma, Juan Liu, Ray Ming

**Affiliations:** 1 College of Life Sciences, FAFU and UIUC Joint Center for Genomics and Biotechnology, Fujian Provincial Key Laboratory of Haixia Applied Plant Systems Biology, Fujian Agriculture and Forestry University, Fuzhou, 350002 Fujian China; 2 Fruit Research Institute, Fujian Academy of Agricultural Sciences, Fuzhou, 350013 Fujian China; 3 College of Agriculture, Fujian Agriculture and Forestry University, Fuzhou, 350002 Fujian China; 4 Department of Plant Biology, University of Illinois at Urbana-Champaign, Urbana, IL 61801 USA

**Keywords:** RNA sequencing, Flowering

## Abstract

The morphogenesis of gynoecium is crucial for propagation and productivity of fruit crops. For trioecious papaya (*Carica papaya*), highly differentiated morphology of gynoecium in flowers of different sex types is controlled by gene networks and influenced by environmental factors, but the regulatory mechanism in gynoecium morphogenesis is unclear. Gynodioecious and dioecious papaya varieties were used for analysis of differentially expressed genes followed by experiments using auxin and an auxin transporter inhibitor. We first compared differential gene expression in functional and rudimentary gynoecium at early stage of their development and detected significant difference in phytohormone modulating and transduction processes, particularly auxin. Enhanced auxin signal transduction in rudimentary gynoecium was observed. To determine the role auxin plays in the papaya gynoecium, auxin transport inhibitor (N-1-Naphthylphthalamic acid, NPA) and synthetic auxin analogs with different concentrations gradient were sprayed to the trunk apex of male and female plants of dioecious papaya. Weakening of auxin transport by 10 mg/L NPA treatment resulted in female fertility restoration in male flowers, while female flowers did not show changes. NPA treatment with higher concentration (30 and 50 mg/L) caused deformed flowers in both male and female plants. We hypothesize that the occurrence of rudimentary gynoecium patterning might associate with auxin homeostasis alteration. Proper auxin concentration and auxin homeostasis might be crucial for functional gynoecium morphogenesis in papaya flowers. These results will lead to further investigation on the auxin homeostasis and gynoecium morphogenesis in papaya.

## Introduction

In angiosperms, 89% species are hermaphrodite, 5% are monoecy, and 6% are dioecy^1^. Male flowers arose due to the occurrence of female sterility (gynoecium abortion) and male fertility in hermaphroditic flower ancestors. It was also evolutionarily benefited from male–female mating strategies to maintain offspring genetic diversity by promoting pollen dispersal in population, although a seemingly futile strategies for agriculture^2^. The transition from hermaphroditic ancestors to male in populations caused by genetic mutations in genomic sequence was common, while reverse to hermaphrodite is rare. The past studies have provided convincing evidence of sex reversal, such as papaya^3,4^.

Gynoecium morphogenesis are resulted from the temporal–spatial interactions of genes (such as homeobox transcription factors) and phytohormones^5^. From previous reports, it can be inferred that the lability in gynoecium morphogenesis was associated with ecological environment or climatic change. The adverse environmental conditions, such as deficit of light, water, or nutrition, often favor maleness in dioecious, androdioecious, and monoecious plants^6–8^. The environmental changes coupled with phytohormones imbalance would easily induce floral organ abortion^9^. All of these have attracted biologists to understand the possible inherited and environmental regulatory mechanism of gynoecium morphogenesis in plants.

Trioecious papaya is an excellent system to explore the genetic and environmental regulatory mechanism for gynoecium development. This tropical fruit crop has three sex forms, which display different features of gynoecium morphogenesis in populations. For wild population, trees are female (functional gynoecium) and male (rudimentary gynoecium). For cultivated varieties, there are predominantly hermaphroditic trees (restorations of rudimentary gynoecium in flower) by removing their female counterpart, because of their economic importance and pollination efficiency. Several studies revealed that XY nascent sex chromosome system control the sex in papaya. The sex chromosome combination in female is XX, while that of male and hermaphrodite is XY and XY^h^, respectively. Hermaphrodite papaya diverged from its male wild papaya ancestors recently, and was a product of human domestication^3^. However, we still do not have a candidate gene disrupting and restoring functional gynoecium development in male and hermaphrodite papaya. Beyond that, it is an unstable system, because the hermaphroditic to male-like flowers or complete reversal of male to hermaphrodite flowers are common under continuous high or low temperature^10^. It is also unclear how the environmental factors affect the target gene or genes resulting in these noticeable changes.

Interestingly, in hermaphroditic papaya, temperature has great influence in gynoecium fertility. In spring, the trees always bear one dominant perfect flower with normal androecium and gynoecium on the top of small inflorescence and few auxiliary/secondary male-like (female sterile) flowers on both sides. While in summer, female sterility occur with rudimentary gynoecium in dominant hermaphroditic flower, while the female papaya exhibits the strong tolerance to summer stress. However, in autumn, with temperature alleviation, the dominant hermaphroditic flowers with normal as well as rudimentary gynoecium initiated simultaneously on individual plants.

Our aim is to identify the regulatory mechanisms related to gynoecium morphogenesis in papaya through the clues of metabolism changes by differential gene expression analysis. Comparing the transcriptome of rudimentary gynoecium with the functional ones, we found that the expression of genes involving in auxin signal transduction was enhanced in rudimentary gynoecium. Weakening the auxin flow is sufficient to restore gynoecium development in male flowers. The study elucidated a genetic and environmental regulatory mechanism in gynoecium morphogenesis for papaya.

## Materials and methods

### Plant material

Gynodioecious papaya variety “Zhongbai” and dioecious papaya variety “Zhonghuang” were planted in the research station of Fujian Agriculrture and Forestry University (FAFU). In November 2015, small inflorescences including rudimentary gynoecium of hermaphroditic dominant and auxiliary flowers, functional gynoecium of hermaphroditic dominant and female flowers from “Zhongbai”, as well as, functional gynoecium of female flowers and rudimentary gynoecium of male flowers from “Zhonghuang” were collected near the trunk apex and preserved in RNA later solution for 2 days. The ambient temperature fluctuated from 20 ˚C at night to 31 ˚C at day for these two days.

To investigate the differentially expressed genes between rudimentary and functional gynoecium, five comparisons were designed. Four comparisons were from “Zhongbai” includes (i)" the functional gynoecium of hermaphroditic dominant flowers and rudimentry gynoecium of auxiliary flowers (H_dom-FG vs. H_aux-RG), (ii) functional gynoecium of hermaphroditic dominant flowers and rudimentary gynoecium of hermaphroditic dominant flowers (H_dom-FG vs. H_dom-RG), (iii) functional gynoecium of female flower and rudimentary gynoecium of hermaphroditic dominant flowers (F-FG vs. H_dom-RG), and (iv) functional gynoecium of female flower and rudimentary gynoecium of hermaphroditic auxiliary flowers (F-FG vs. H_aux-RG), while, one was from dioecy variety “Zhonghuang” (functional gynoecium of female flower and rudimentary gynoecium of male flower (F.FG vs. M.RG)).

We selected early stage flowers with length <2 mm and separated functional and rudimentary gynoecium by anatomic dissection under microscope for RNA extraction. Three biological replications for each type of sample were collected with each replication of 5–30 flowers.

### RNA isolation, library preparation, and high-throughput sequencing

Total RNA was extracted using RNA Isolation Tripure reagent (Roche, Cat.No.11667001) and RNA-seq libraries were prepared using Ultra RNA Library Prep Kit (NEB, #E7770L) according to the manufacturer’s instructions. High-throughput sequencing of indexed libraries was done on the Illumina Hiseq^TM^2500 system to obtain 100-nt pair-end reads in 2016. Low quality reads with Poly-N and adapter sequence were filtered out using Trimmomatic script with default parameter values^11^.

### DEGs(differentially expressed genes), gene ontology, and metabolic pathways enrichment analysis

Paired-end reads were aligned to the papaya reference genome (Cpapaya_113_ASGPBv0.4 from Phytozome, https://phytozome.jgi.doe.gov/pz/portal.html) by STAR Aligner 2.6^12^. Transcriptome assembly and DEGs analysis were conducted by Cufflinks 2.21^13^ following the official manual (http://cole-trapnell-lab.github.io/cufflinks/).

Gene ontology enrichment and metabolic pathways enrichment analysis were performed by Plantregmap^14^ and KOBAS 3.0 web server^15,16^.

The dynamic pattern of differentially expressed genes related to phytohormone signal transduction was presented by R package “pheatmap” according to the corresponding converted value (mean = 0 and standard deviation = 1), which was centered and scaled from FPKM numeric values of different samples using “pheatmap” package parameter “scale”.

### Exogenous auxin and auxin transport inhibitor application on papaya

Six-months-old “zhonghuang” papaya plants grown on FAFU campus were chosen. After appearance of small inflorescences, auxin transport inhibitor (NPA) and synthetic auxin analogs (NAA and 2,4-D) were sprayed towards the trunk apex which produced inflorescences on the junction of stem and leaf petiole. The concentrations of NPA was set to 10 mg/L, 30 mg/L, and 50 mg/L respectively, while that of NAA and 2,4-D were 1 mg/L and 10 mg/L for both. For each treatment, four biological replications were used. To enhance application efficiency of synthetic auxin analogs and auxin transport inhibitor, application interval was set to spray once after every two weeks for 8 weeks from September to November 2017.

## Results

### Identification of DEGs comparing rudimentary and functional gynoecium

12.1–21.7 million raw reads per library from gynodioecious papaya var. “Zhongbai” and 13.1–18.3 million raw reads per library from dioecious papaya var. “Zhonghuang” were generated. After removing low quality reads, the clean reads were mapped to papaya draft genome by STAR aligner. The reads uniquely mapped to genome accounted for 81.3–87.2% of clean reads (Supplementary Tab. 1).

**Fig. 1:**
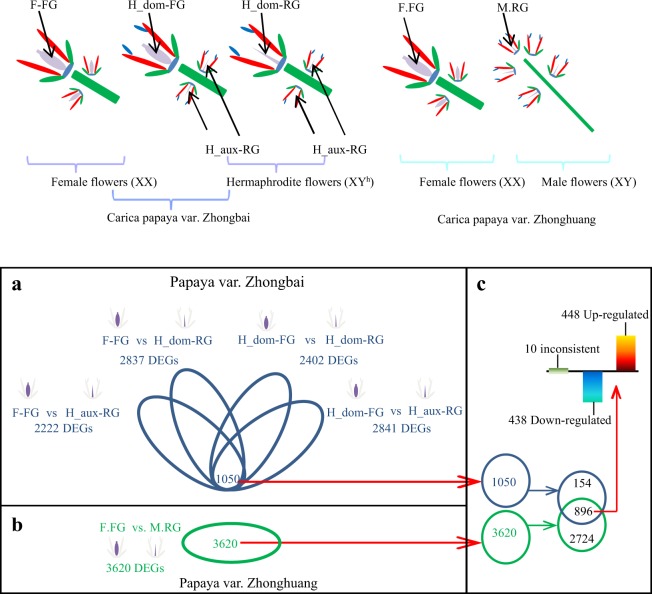
DEGs in the comparisons of gynoecium samples from different flowers of papaya (hermaphrodite, male and female). **a** DEGs from female and hermaphrodite flowers of “Zhongbai” (**b**) DEGs from male and female flowers of dioecious variety “Zhonghuang” (**c**) Key common DEGs from both “Zhongbai” and “Zhonghuang” rudimentary gynoecium

To prevent potential differences of gene expression pattern from different genetic background, we normalized and calculated gene expression values in FPKM (fragments per kilobase of exon per million fragments mapped) when compared the gynoecium samples from papaya variety “Zhongbai”. The functional gynoecium of hermaphroditic dominant flowers and female flowers were considered as reference (baseline) when comparing against the rudimentary ones. Using female functional gynoecium as reference, 2837 genes expressed differently between F-FG and H_dom-RG, while comparing F-FG with H_aux-RG, 2222 genes were differentially expressed. Using functional gynoecium of hermaphroditic dominant flowers as reference, 2402 and 2841 DEGs were identified while comparing H_dom-FG against H_dom-RG and H_aux-RG, respectively. In total, 1050 common DEGs were found among these four types of comparisons (Fig. 1a).

The same approach was used to analyze samples from male and female flowers of dioecious variety “Zhonghuang”. We found 3620 DEGs from the comparison of functional gynoecium of female with rudimentary gynoecium of male flower (Fig. 1b).

Interestingly, we found 896 key common DEGs when compared 1050 common DEGs from “Zhongbai” and 3620 DEGs from “Zhonghuang”. Out of 896, 448 DEGs were upregulated while 438 were downregulated in both “Zhongbai” and “Zhonghuang” rudimentary gynoecium samples. While, 10 DEGs showed inconsistent expression pattern between these two varieties and were neglected (Fig. 1c).

**Fig. 2:**
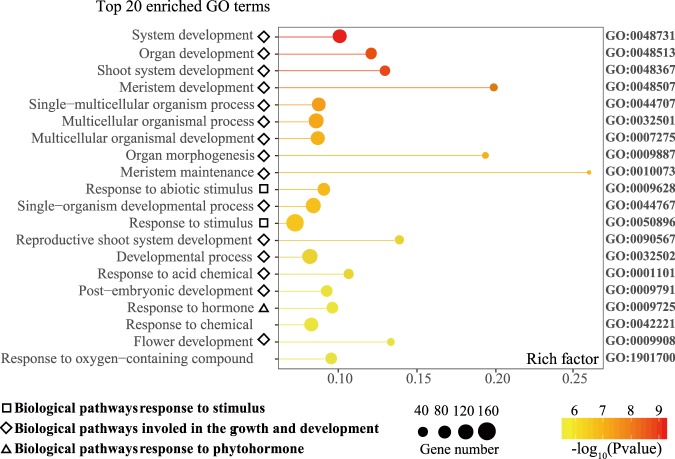
GO enrichment analysis of 896 key common DEGs

To further explore the differences in metabolism and development which associated with phenotypic sex character, we investigated the biological roles of 896 key DEGs using GO annotation and enrichment analysis. The results showed that the most abundant 20 GO terms of 896 key DEGs were mainly classified into three biological pathways categories (Fig. 2), i.e., response to stimulus (indicated by square), the growth and development (by rhombuses), and response to phytohormone (by triangle).

### Metabolic pathways enrichment analysis revealed differences in phytohormone signal transduction

Based on the corresponding homologous protein of Arabidopsis by protein sequence similarity Blast searches, we conducted the KEGG enrichment analysis for 896 key DEGs. The results suggested that the plant phytohormone signal transduction was the pathway of most significant enrichment (corrected *P*-value 3.15e-07) in the comparisons among functional and rudimentary gynoecium samples, while the other pathways such as phenylpropanoid biosynthesis and secondary metabolites biosynthesis had *P*-value > 0.001. So the phytohormone signaling might play a critical role in transitioning from normal gynoecium to rudimentary gynoecium.

**Fig. 3:**
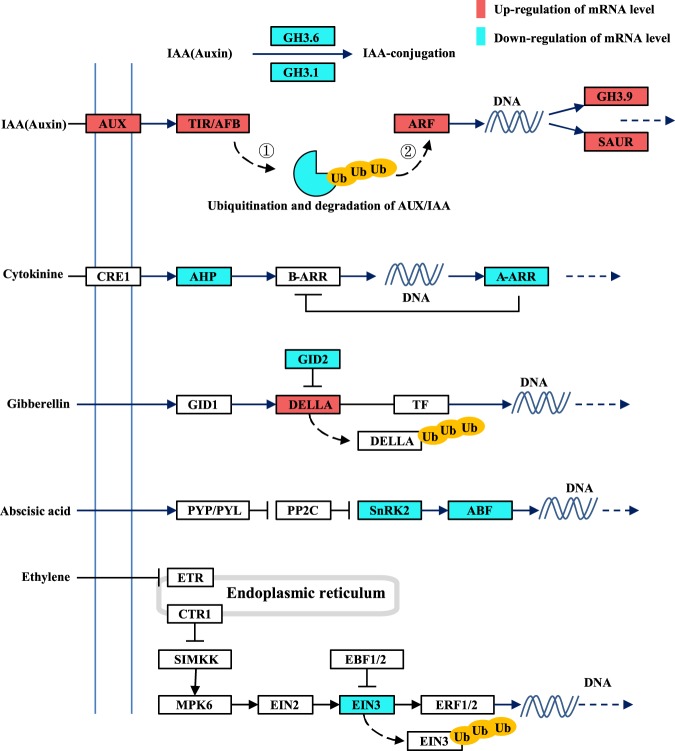
KEGG analysis emphasized the differential gene expression of phytohormone signaling transduction in rudimentary gynoecium compared to functional gynoecium, red boxes indicate upregulated gene, cyan boxes indicate downregulated gene, Ub in yellow circles show the degradation of signaling molecule by the ubiquitin-proteasome, ①TIR1/AFB complex promoted ubiquitination and degradation of Aux/IAA, ②degradation of Aux/IAA released the transcript repression of ARF

To get a better identification on the major phytohormone signal transduction regulating the phenotypic development of different gynoecium, DEGs were represented in corresponding signaling pathways (Fig. 3). There was a clearly defined enrichment in auxin signal transduction. In TIR1/AFB-Aux/IAA-ARF auxin signaling pathway, TIR1/AFB (TRANSPORT INHIBITOR RESPONSE1/AUXIN SIGNALING F-BOX PROTEIN) is a core subunit of SCF multi-protein E3 ubiquitin ligase complex, and acts as an intracellular receptor. AUX1 encodes a specific membrane-bound auxin influx transporter. In all of rudimentary samples, increased *TIR1/AFB* transcript expression was found to be concomitant with the accumulating *AUX1* transcript expression, in response to extracellular auxin stimulus. Aux/IAA(AUXIN/INDOLE ACETIC ACID, auxin response factors transcriptional repressors) genes showed decreased expression pattern in these rudimentary samples, which maintained Aux/IAA protein content at a relatively low level in the cells and further promote the transcription of ARF(AUXIN RESPONSIVE FACTORS) and ARF-mediated early auxin response genes (SAURs and GH3.9) as well as increase the auxin effect. And the decrease in transcript expression of some GH3s involving in auxin conjugation, such as GH 3.1 and GH3.6, suggested there would be more accumulation of active auxin conferring a continuous auxin stimulus in rudimentary gynoecium. Besides that, PIN transcription was downregulated (Supplementary Tab. 2), which might cause further auxin accumulation and affect signal transduction.

**Fig. 4:**
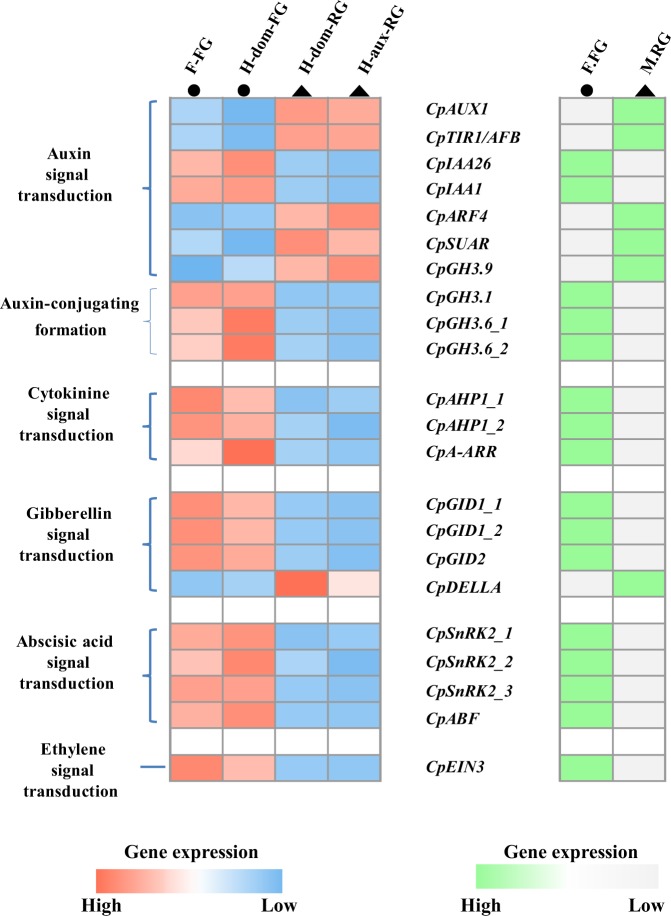
Similar pattern of gene expression of related phytohormone responsive signaling transduction factors in comparison between functional and rudimentary gynoecium samples

Further investigation on the changes of DEGs in phytohormone signal transduction was made by presenting heatmaps (Fig. 4, gene symbol, gene id, and FPKM values were provided in Supplementary Tab. 2). For five signaling transduction, the crucial and indicative phytohormones responsive factors showed similar pattern of gene expression, which could be interpreted as similar phytohormone regulation or response when comparing rudimentary and functional gynoecium in papaya.

### Weakening of polar auxin transport result in female fertility restoration in male flower

RNA-seq analysis results underlined the potential accumulation of active auxin in rudimentary gynoecium. To observe the effect of auxin in distinguish gynoecium tissue, auxin transport inhibitor (NPA) and synthetic auxin analogs (NAA and 2,4-D) were sprayed towards the trunk apex of male and female plants of diecious variety Zhonghuang.

**Fig. 5:**
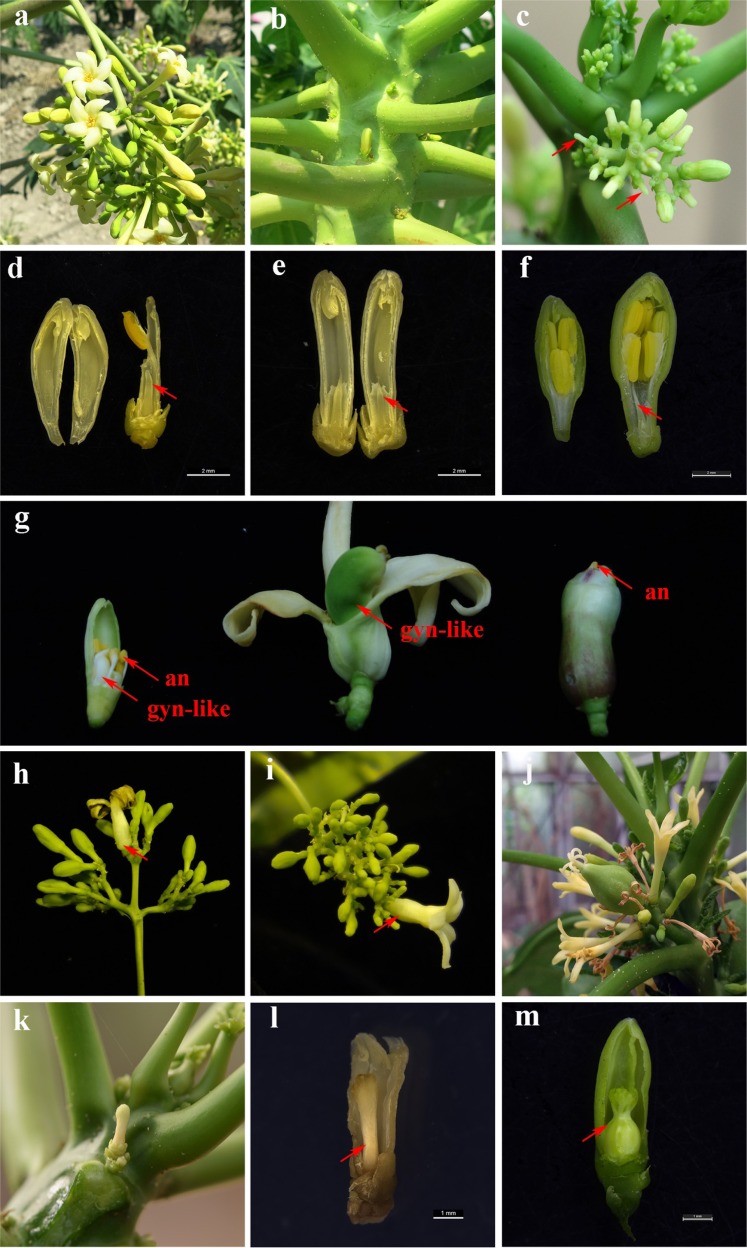
The phenotypic changes of the NPA-treated flowers in dioecious papaya “Zhonghuang”. **a** Male inflorescences on the male plants. **b** Female flowers on the female plants. **c** Male inflorescences treated by NPA. Arrow indicated a long-rod phenotype flowers due to a decrease in androecium number. **d** A long-rod phenotype male flower with only one androecium. Arrow indicated the rudimentary gynoecium. Bar = 2 mm. **e** A long-rod phenotype male flower with no trace of filaments and anthers. Arrow indicated the rudimentary gynoecium. Bar = 2 mm (**f**) Normal male flower. Arrow indicated the rudimentary gynoecium. Bar = 2 mm. **g** Phenotypically similar perfect hermaphrodite flowers with fertile gynoecium in male plant. Gynoecium-like structures (gyn-like) bore flat and smooth stigmas(left), bore feathery stigmas (mid), and entirely embedded in merged outer whorls of floral system(right). All newly induced hermaphrodite flowers referred to have visible androecium(an). **h,i** The transformed primary male flowers surrounded with adjacent clusters of male flowers. Arrow indicated the transformed primary male flowers. **j** The inflorescences of *Vasconcellea monica*. **k,l** The NPA-treated female flower bore a stalk-like gynoecium with stigma. Arrow indicated the stalk-like gynoecium, Bar = 1 mm (**m**) Normal female flower. Arrow indicated the functional gynoecium, Bar = 1 mm

10 mg/L polar auxin transport inhibitors (NPA) application on papaya shoot apex resulted in striking changes in morphological feature of male papaya. Some long-rod male flowers appeared in male trees first (Fig. 5c), which later demonstrated that it was a result of the decrease in androecium numbers (<10) (Fig. 5d). We collected growing inflorescences separately from four male trees to investigate the development of androecium in 100 male flowers, of which 8%, 12%, 18%, 22% were identified as male flowers with no traces of filaments and anthers but rudimentary gynoecium (Fig. 5e).

Some phenotypically similar perfect hermaphrodite flowers with fertile gynoecium appeared, surrounding with adjacent clusters of normal male flower with 10 anthers. In these sexual transformed flowers, some gynoecium-like structures bore flat and smooth stigmas, some bore feathery one, and some were entirely embedded in merged outer whorls of floral system, but all newly induced hermaphrodite flowers referred to have visible androecium (Fig. 5g). For most cases, increasing size of stout base of young flower buds on NPA-treated male plant mean gynoecium-like structures had simultaneously protruded out of the receptacle (Fig. 5h, i).

The arragement of newly appeared sexually transformed flowers in NPA-treated male papaya was similar to that of *Vasconcellea monica* (the only monoecious specie in Caricaceae) inflorescent with female flowers surrounded with adjacent male flowers (Fig. 5j).

The peduncle length of the NPA-treated plants was much shorter than that of control male plants. All these present observations clearly suggested that the small number of hermaphrodite-like flowers start appearing in male plants after NPA treatment.

However, female plants did not show significant changes in 10 mg/L NPA treatment. Only few flowers on female trees bore a stalk-like gynoecium (Fig. 5k). It might happen because of critically low level of auxin concentration due to blockage of auxin transporter proteins, same phenotype was observed in Arabidopsis pin/pid mutants^17^. The phenotypic character of stalk-like gynoecium organ (Fig. 5l) caused by NPA spraying in female was found to be different from rudimentary gynoecium in male flower or hermaphroditic auxiliary flowers.

The restored female fertility in NPA-induced papaya plants was concentration-dependent. The suitable NPA concentration (10 mg/L) caused the formation of hermaphrodite-like flower in male trees while higher NPA concentration of 30 and 50 mg/L resulted in more deformed flowers in both male and female plants, which showed abnormal differentiation of gynoecium organs. It suggested that severe auxin transport disruption would abolish gynoecium morphogenesis. Therefore, a proper auxin concentration is necessary for gynoecium morphogenesis.

After the cessation of NPA treatment, the normal inflorescences gradually restored in the apical shoot as expected. The induced foliage fusion was the adverse effect of long-term NPA treatment (Supplementary Fig. 1).

On the other hand, NAA and 2,4-D (synthetic auxin analogs) directly sprayed to male and female individuals could not affect the inflorescence morphologies, although small and deeply pinnate lobed leaves arose.

## Discussion

The highly differentiated morphology of gynoecium in different sexual flower of triecious papaya is determined by genetic and environmental factors, but the regulatory mechanism was unclear. In this study, differential gene expression pattern between functional and rudimentary gynoecium revealed that gynoecium morphologenesis and development are associated with three main biological processes, including (i) response to stimulus which may induce abnormalities, unbalanced phytohormone distribution, ectopic transcription factor modulation, and aberrant cell proliferation growth; (ii) response to growth and development, which may be associated with female gametophyte and fertilization-independent development; and (iii) reponse to phytohormones. Of these, the phytohormone responsive DEGs seem to be more critical. KEGG enrichment of DEGs implied that phytohormones signaling transduction was the most significant enrichment pathway when compared functional and rudimentary gynoecium. More importantly, auxin-related DEGs were all highlighted in known signal transmission processes, suggesting that auxin had a much greater influence than other hormones on regulation of gynoecium morphogenesis.

Conversion from functional gynoecium to rudimentary gynoecium in papaya seems to be related to over-accumulation in auxin, instead of deficiency in auxin. In rudimentary gynoecium samples, the mRNA levels of auxin influx carrier AUX1, auxin receptor TIR were upregulated, coupling with increased transcription of auxin response genes like *SAURs* and *GH3s*, which might be triggered by accumulation of extracellular auxin. And the decrease in transcript expression of gene participated in auxin conjugation and efflux will further keep up a consistently high content of active auxin in cells. The same situation occurs in rudimentary gynoecium of male and hermaphrodite flowers, when compared to the one of female flowers and perfect hermaphrodite flowers. It was therefore suggested that auxin accumulation might be associated with gynoecium morphogenesis anomalies. This was confirmed by exogenous NPA application through weakening of polar auxin flow in male flower which partially restored the defects of gynoecium development.

The phytohormone auxin plays a crucial role in organs morphogenesis. Recent reports have unraveled that morphogenetic processes of flower and leaf primordium occurred in the certain site of peripheral zone rather than the central undifferentiated cells and waiting meristem cells in shoot apical meristem tissue^18–21^. In these positions, auxin temporal-spatial distribution and homeostasis, including the orientation, timing and pattern of auxin maxima or minima, presage the new organs primordium formation and guide the further morphogenesis through pre-patterning^22–27^. Deficiency in auxin signaling and transport could influence gynoecium development. In our experiment, with excessive application of NPA, severe blocking of the auxin stream in shoots of female papaya made few flowers bore a stalk-like gynoecium with stigma. This phenotype appeared to resemble to Arabidopsis mutants which were deficit in auxin signaling and transport. Attenuation of auxin signaling in *ETT* (*ARF3* mutant) caused an expansile basal gynophore, stylar and stigma, as well as diminished ovary valves, like a thin stalk topped with stigma anlages^28–30^. Disruption of polar auxin transportation in PIN (auxin efflux carrier) and PID (a kinase phosphorylate PIN and direct PIN’s distribution) mutant resulted in stalk-like gynoecium^17^. Stalk-like gynoecium caused by disruption of polar auxin transport is different from rudimentary gynoecium in male flowers or hermaphroditic auxiliary flower of papaya. NPA-meditated deficit in auxin transport and signaling only made the female flowers with functional gynoecium bear stalk-like gynoecium structure. But, in contrast, after weakening auxin transport and signaling, female restoration of male flowers has proved the necessity of decreasing auxin transport and signaling in sex reversal of male to hermaphrodite flowers.

Our observation of the differences between functional gynoecium and rudimentary gynoecium in hermaphroditic flowers after the hot summer help us underline the environmental effects on gynoecium development. As far as we know, auxin was not only involved in organs morphogenesis, but also discovered to be implicated in the response and resistance against unfavorable environment. Many studies were reported that auxin accumulation in Arabidopsis seedlings especially for adverse conditions, such as high temperature and vegetative shading. An accumulation in auxin production resulted in elongation of hypocotyl and leaf petiole while seedling grew in elevated temperature or shade conditions^31,32^. So the excessive auxin may contribute to gynoecium morphogenesis anomalies in hermaphroditic flower under high temperature conditions, considering that floral organs have evolutionarily derived from leaves.

**Fig. 6:**
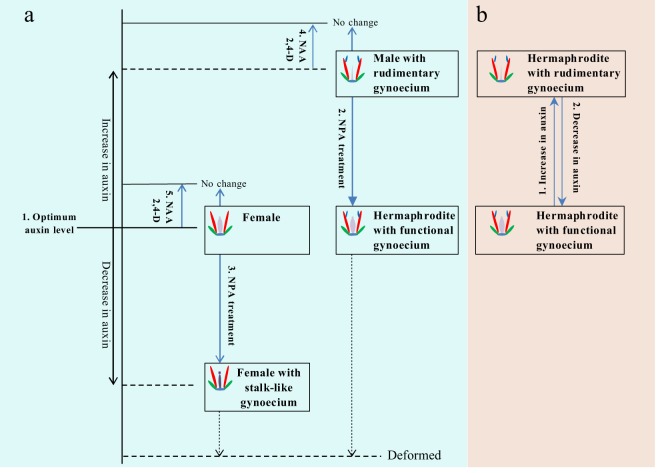
Model shows how auxin affects the gynoecium morphogenesis in papaya. **a** 1. Optimal level of auxin is critical for functional gynoecium development 2. Auxin transport inhibitor (NPA) treatment to male flower resulted in polar auxin flow weakening that might drop the auxin concentration to critical level for functional gynoecium development and result in sex reversal from male flower to perfect hermaphrodite flower 3. NPA application to female flower resulted in critically low auxin concentration with the development of stalk-like gynoecium structure (non-functional) completely different from rudimentary gynoecium 4 & 5. A smaller increase in auxin concentration under auxin analogs application resulted in no changes in both male and female flowers. **b** Hermaphrodite flowers are easily influenced by environment (temperature) comparing to both male and female 1. In summer, increased temperature may result in increased auxin level with the development of rudimentary gynoecium 2. While, in autumn, decrease in temperature may cause the development of functional gynoecium due to drop in auxin level to the critical level for functional gynoecium development

Based on our transcriptional expression analyses of auxin-related signaling genes and experiments using auxin transport inhibitor, we proposed a hypothetical model that auxin homeostasis determines the highly differentiated morphology of gynoecium in papaya flowers. In our model (Fig. 6), an optimal level of auxin was necessary for the correct morphogenesis of functional gynoecium. The occurrence of rudimentary gynoecium in male flower might be caused by excessive accumulation of auxin, and the female fertility restoration in NPA-induced male-to-hermaphrodite transformed flowers is likely due to weakening of polar auxin flow to the optimum level critical for functional gynoecium development. For female flowers, the same NPA treatment, reducing endogenous auxin content to critically low concentration, led to the emergence of stalk-like gynoecium. As the spray concentration increases, severe blockage of auxin transport under excessive NPA application (very low auxin) ultimately resulted in deformed flowers in both male and female.

In hermaphrodite flowers, auxin homeostasis alteration can also explain the conversion from functional gynoecium to rudimentary gynoecium. It was reported that, for gynodioecious variety “Sunrise”, the endogenous auxin contents in hermaphrodite flowers with rudimentary gynoecium was highest while that in female and hermaphrodite flowers with functional gynoecium were lower^33^. Under the same genetic background, high concentration of auxin in hermaphrodite flowers coincides with the phenotype of rudimentary gynoecium, suggesting that auxin homeostasis alteration have a significant impact on gynoecium morphogenesis in hermaphrodite flowers. Given that high temperature has induced auxin accumulation in Arabidopsis seedlings, we infer that excessive auxin accumulation caused by temperature change led to rudimentary gynoecium.

Our hypothetical model summarizes all the findings in this study and emphasizes the influence of auxin homeostasis on gynoecium morphogenesis in papaya flowers of different sex types. If this hypothesis is correct, we are inclined to believe that it is a gene on the Y chromosome that caused excessive auxin accumulation in male flower, and the increase of auxin content triggered by unfavorable environmental conditions might result in the rudimentary gynoecium in hermaphrodite flower.

In conclusion, the transcriptome analysis and NPA treatment demonstrated that auxin played an important role in papaya gynoecium morphogenesis. The results showed that proper auxin concentration and auxin homeostasis were necessary for functional gynoecium development. However, the gynoecium tissue is pretty small at the early flower stage and arduous to separate, making it difficult to collect enough samples acquired for endogenous auxin content measurement. Therefore, our next step is to detect its auxin content in flowers of different sex types, especially in flowers of male-to-hermaphrodite sex reversal papaya mutants reported in our previous research^34^, to prove our hypothesis.

## Supplementary information

Supplemental table and figure

## Data Availability

Raw datas of high-throughput sequencing were deposited in NCBI SRA (SRA accession: PRJNA549650, https://www.ncbi.nlm.nih.gov/sra/PRJNA549650).
