# Supplementary material for: Auxin regulation involved in gynoecium morphogenesis of papaya flowers
Source: Hortic Res. 2019 Nov 1;6:119. doi: 10.1038/s41438-019-0205-8 (PMC6823548; doi:10.1038/s41438-019-0205-8)
Supplement: Supplementary file 1 — Supplemental table and figure [file 41438_2019_205_MOESM1_ESM.pdf]

Supplementary Tab. 1 Statistics summary of different flower samples

Variety	Sex	Sources	Samples	Reads number	Uniquely mapped reads number	Uniquely mapped reads %
Zhongbai	Female flowers		F-FG-1	14818720	12479097	84.2%
			F-FG-2	18910565	16157509	85.4%
			F-FG-3	17757504	14946366	84.2%
	Hermaphroditic flowers	Dominant flowers	H_dom-FG-1	18445542	15288277	82.8%
			H_dom-FG-2	19970646	17241378	86.3%
			H_dom-FG-3	20164217	16385653	81.3%
			H_dom-RG-1	17002622	14606595	85.9%
			H_dom-RG-2	21753451	18950347	87.1%
			H_dom-RG-3	12145477	10014029	82.4%
		Auxiliary flowers	H_aux-RG-1	18699560	16225050	86.8%
			H_aux-RG-2	18412657	15168292	82.4%
			H_aux-RG-3	16272813	14065551	86.4%
Zhonghuang	Female flowers		F.FG-1	15542782	13556652	87.2%
			F.FG-2	15941598	13890426	87.1%
			F.FG-3	17768971	15291339	86.1%
	Male flowers		M.RG-1	13157727	11310929	86.0%
			M.RG-2	15510777	12923914	83.3%
			M.RG-3	18367834	15880162	86.5%

Supplementary Tab. 2 FPKM of related signaling transduction molecule gene in the comparisons between functional and rudimentary gynoecium samples

**Biological process**	**Gene symbol**	**Gene ID**	**FPKM**					
			F-FG	H_dom-FG	H_dom-RG	H_aux-RG	F.FG	M.RG
Auxin signal transduction	*CpAUX1*	(evm.TU.supercontig_19.209)	78.4505	64.0130	133.4070	126.8460	53.5650	87.5300
	CpTIR1/AFB	(evm.TU.supercontig_27.130)	34.9850	30.6249	53.3262	52.6555	41.3501	66.5562
	CpIAA26	(evm.TU.contig_35052.1)	145.1840	169.6370	57.2707	47.8887	249.7960	96.0987
	CpIAA1	(evm.TU.supercontig_87.29)	66.6903	70.05450	31.8663	28.4595	69.0893	36.4540
	CpARF4	(evm.TU.supercontig_139.80)	59.1576	71.6908	267.4030	315.1410	105.6460	406.7520
	CpSUAR	(evm.TU.supercontig_2619.1)	80.2177	42.5502	198.4090	170.7930	56.5522	183.7190
	CpGH3.9	(evm.TU.supercontig_9.204)	41.6617	58.2735	94.8120	105.5990	33.8957	142.8410
Auxin conjugating formation	*CpGH3.1*	(evm.TU.supercontig_6.74)	18.3295	18.4227	1.5203	1.4177	10.4661	1.89726
	CpGH3.6_1	(evm.TU.supercontig_1065.2)	12.1548	16.6130	3.86713	2.8183	22.4035	6.48987
	CpGH3.6_2	(evm.TU.contig_32826.1)	20.5534	28.1409	9.0444	6.7900	39.0433	13.6209
Auxin polar transport	*CpPIN1a*	(evm.TU.contig_35483.1)	104.3550	107.0910	65.6750	52.3855	132.0590	68.6681
	CpPIN1b	(evm.TU.contig_44255.1)	104.1900	107.8910	70.9593	63.4071	199.8260	87.7924
Cytokinine signal transduction	*CpAHP1_1*	(evm.TU.supercontig_84.120)	174.1290	136.2980	25.0568	36.9384	205.5860	14.3845
	CpAHP1_2	(evm.TU.supercontig_16.84)	336.8710	305.8490	152.2580	118.7420	354.9870	151.4080
	CpA-ARR	(evm.TU.supercontig_64.98)	14.8845	23.5326	5.3995	4.2360	12.1785	0.761709
Gibberellin signal transduction	*CpGID1_1*	(evm.TU.supercontig_84.118)	326.5290	295.0140	161.2040	151.4900	325.1100	178.5630
	CpGID1_2	(evm.TU.supercontig_731.1)	201.5400	178.4570	92.8497	85.4265	211.2540	103.6090
	CpGID2	(evm.TU.supercontig_21.140)	257.3810	242.6050	131.8800	117.6840	350.7620	177.3180
	CpDELLA	(evm.TU.supercontig_75.88)	28.3233	31.9491	82.6978	55.3735	20.0456	39.5802
Abscisic acid signal transduction	*CpSnRK2_1*	(evm.TU.supercontig_98.59)	56.4413	60.7926	24.9977	26.3229	106.5280	18.7974
	CpSnRK2_2	(evm.TU.supercontig_62.55)	263.3710	313.0220	148.0030	117.0400	362.2200	233.6430
	CpSnRK2_3	(evm.TU.supercontig_36.167)	27.7257	27.8681	7.55427	5.9136	36.1018	11.9450
	CpABF	(evm.TU.supercontig_5.28)	38.6877	45.3772	3.2103	1.8227	81.2703	3.0083
Ethylene signal transduction	*CpEIN3*	(evm.TU.supercontig_54.30)	21.4569	17.9688	8.15673	8.0689	65.8606	14.2208

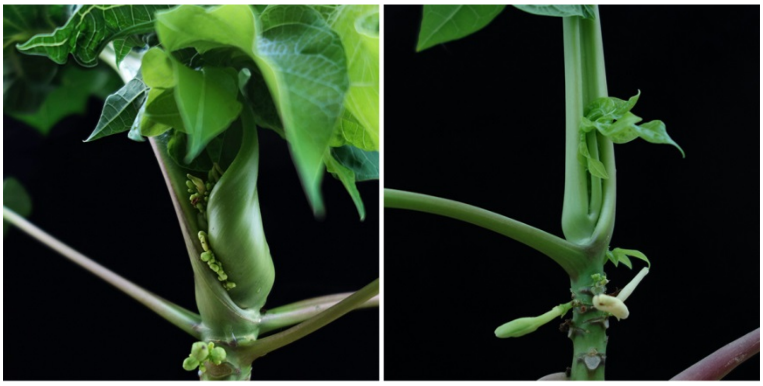

Supplementary Fig. 1 The side effect of long-term NPA treatment (male papaya)
